# Obituary: professor sir Patrick Bateson FRS and dog welfare

**DOI:** 10.1186/s40575-017-0048-2

**Published:** 2017-09-26

**Authors:** David Richard Sargan

**Affiliations:** University of Cambridge, Cambridge, Cambs UK

Professor Sir Patrick Bateson, who died on August 1st 2017, at the age of 79, was a cultured and amusing but persuasive debater and writer: a good man to have at your side in an argument, but also a gentle yet effective interrogator. He was also an extremely distinguished zoologist: past President of the Royal Zoological Society of London (2004–2014), Biological Secretary of the Royal Society (1998–2003), and provost of Kings College Cambridge (1988–2003), where he took a particularly keen interest in the gardens.

But his greatest energies were spent on his studies of animal behaviour and within this area he was extremely influential both in our understanding of the development and neurobiology of learning and behavior and subsequently in assessment of animal welfare, where he developed measurements of pain and suffering and then the frameworks in which they could be applied. Initially his interest was in medical research, where “Bateson’s cube” shows us how to take into account the level of animal suffering a piece of research might cause, the certainty of medical benefit, and the quality of research performed (how certain is it that results will be found). Subsequently this expertise was applied to more general areas of animal welfare, so that he led an inquiry financed by the UK National Trust into stag hunting that caused the immediate banning of stag hunting with hounds on their property, and subsequently influenced a UK Government Inquiry whose recommendations led to the ban on hunting deer with dogs. Patrick’s methods were those of careful measurement and observation, minute dissection of the evidence obtained, and direct and simple presentation of his conclusions.

This expertise both in welfare and its measurement, and in development and biology made Patrick a natural choice to consider problems within dog breeding in the wake of the BBC documentary, “Pedigree Dogs Exposed”. After agreeing to a joint invitation from DEFRA, The Kennel Club and the Dogs Trust and their agreeing to his total independence to form and publish his own conclusions, Patrick set about this inquiry with the help of Heather Peck as administrator, ideas-tester and amanuensis. For him this took up the great part of his calendar year 2009. Although the resulting written report is only 41 pages long (excluding prefaces, summary and appendices) it covers a great deal of ground from the domestication events that may have initially influenced how breeds developed, through means of measuring welfare, the theoretical problems of inbreeding (including loss of hybrid vigour and enrichment for recessive diseases), and the real current problems of large scale commercial breeding including those of puppy mills, inherited health problems, and problems connected with exaggerated conformations. Each problem for dogs is described, and then methods for tackling it sought from experts both amateur and professional, road-blocks identified and recommendations made. He concluded with a number of recommendations: the formation of an “Advisory Council on Dog Breeding” to address specific issues both within individual breeds and in dog breeding practices; the collection of prevalence data; the revision of breed standards; the upgrading of the KC Accredited Breeder scheme; a shift in veterinary practice towards preventative medicine; upgrading of requirements for inspection of premises where breeders require licences, including inspection by veterinarians; the introduction of compulsory microchipping; compulsory extensions to DNA based testing for some diseases; and also public education campaigns on the buying and ownership of dogs.

So how successful has Patrick’s work been on behalf of dogs? Given that the publication took place in a world of newly tight money where successive Governments found other things preoccupying them, I think it has been pretty useful. Microchipping of all dogs is a reality. An advisory council did develop recommendations on specific reforms to commercial dog breeding conditions, reforms to some breed standards, and reforms to the KC’s Accredited Breeder scheme - these have been partially taken up, and the KC have adopted more expert advice. The need for more research has been answered by several of the welfare charities in their choice of projects to fund. The means for collecting prevalence data have continued to develop. There are many remaining problems with individual breeds but these are being chipped away at. An initial period where most in the field thought that the lack of funding meant public education was close to impossible, has been replaced by increasing optimism, as ways have been found to approach commerce directly, to use social media cheaply, and to interest broadcasting media without paying directly for the content. There is plenty still to do, but quite a lot to be positive about. So Patrick’s work on dogs has proved one of his many important legacies in a life exceptionally well-lived.

David Sargan 07.08.17.

Editor’s reply.

We are grateful to Dr. David Sargan for providing this informative and sensitive obituary for Professor Sir Patrick Bateson. I (Bill Ollier) was one of the many individuals interviewed by Patrick in 2009 for the ‘Bateson Inquiry’ and can testify to both his thoroughness and the ‘open-minded’ way he approached all the issues involved. In addition we can also trace back the origin of this Journal to the Bateson Inquiry, as a further consequence was an appreciation of the paucity of scientifically robust genetic and epidemiology publications relating to dog breeding and its impact on health and welfare. An obvious consequence was the formation of an international journal dedicated to canid genetics, epidemiology and evolution that was ‘open source’ to all readers and with summaries also suitable for the general population.

On behalf of the editors.

Bill Ollier.

Lorna Kennedy.

